# Endoscopic ultrasonography guided cutting scar of esophageal stricture after endoscopic injection sclerotherapy

**DOI:** 10.1186/s12876-022-02420-9

**Published:** 2022-07-15

**Authors:** Fulong Zhang, Jing Xu, Yuandong Zhu, Yan Shi, Bo Wu, Hai Wang, Chaojun Huang

**Affiliations:** Department of Gastroenterology, HangZhou Xixi Hospital, 2 Hengbu Street, Xihu District, Hangzhou, 310032 China

**Keywords:** Endoscopic ultrasonography, Esophageal stricture, Sclerotherapy

## Abstract

**Objective:**

To investigate efficacy and safety of endoscopic ultrasonography (EUS) guiding to cut the scar of esophageal stricture after endoscopic injection sclerotherapy (EIS).

**Methods:**

The data of 10 patients with oesophageal stricture after esophageal varices EIS in our hospital from September 1, 2021 to December 31, 2021 treated by cutting the scar guided by ultrasonic endoscopy were retrospective, and the efficacy was evaluated.

**Results:**

The dysphagia was obviously relieved in 9 patients during follow-up, and 1 patient suffered dysphagia again after the treatment. There was no complications of perforation, bleeding and infection among the paitents.

**Conclusion:**

EUS guiding to cut the scar of esophageal stricture after EIS was safe and reliable.

**Supplementary Information:**

The online version contains supplementary material available at 10.1186/s12876-022-02420-9.

## Introduction

Oesophageal stricture is one of the complication of EIS [1]. It is related to local inflammation, ulceration, and fibrosis caused by multiple EIS [2, 3]. The main symptom of oesophageal stricture is dysphagia. No standard treatment for oesophageal stricture after EIS is mentioned in international guidelines or consensuses [4–7]. Furthermore, patients often have a prolonged prothrombin time and thrombocytopenia, and balloon dilation for stricture complicated by EIS for oesophageal varices has poor outcomes [8]. We want to investigate efficacy and safety of EUS guiding to cut the scar of esophageal stricture after EIS in this article.

## Methods

### Object

We reviewed the data of 10 patients with oesophageal stricture after EIS in our hospital from September 1, 2021 to December 31, 2021 treated by cutting the scar under EUS guidance.

### Inclusion criteria

(1) According to the Stooler classification [9, 10], the degree of oesophageal stricture was grade II–VI. (2) The diameter of the oesophageal cavity was more than 2.6 mm, so an ultrasonic endoscope could pass. (3) The oesophageal stricture was not treated with surgery.

### Exclusion criteria

(1) Severe coagulation disorder (prothrombin time > 17 s); (2) unstable blood pressure (systolic blood pressure < 80 mmHg or > 160 mmHg); (3) heart failure (Ejection Fraction < 50%), lung failure (PaO2/Fi O2 < 300 mmhg), or other organ failure; and (4) oesophageal malignancy.

### Follow-up

The patients were followed up between three to six months: (1) The symptoms would be consultated once a week through the cell phone.We would record the complication (perforation, bleeding, and infection), and degree of the dysphagia of oesophageal stricture [9]. (2) The endoscopy would be performed every three months after treatment.

### Assessment

According to the Stooler criteria, degree of oesophageal stricture and dysphagia [9, 10]: Grade 0: There was no dietary restriction. Grade I: Soft food could be eaten smoothly, and food whose diameter is more than 13 mm could be passed. Grade II: Semi-liquid food whose diameter was between 8 and 13 mm could be passed. Grade III: Only liquid diet whose diameter was among 3 to 8 mm; Grade IV: Liquid diet with particles less than 3 mm in diameter was difficult to pass.

The relief criteria of oesophageal stricture were as follows: (1) The degree of stricture recovered to grade 0–I. (2) The body of the standard gastroscope could pass through the oesophagus without any resistance, and the diameter of the gastroscope was 1 cm. (3) There was no stricture recurrence during the 8-week follow-up [11].

The criteria for stricture recurrence were as follows: (1) The degree of stricture worsened to grade II–IV again. (2) The body of the standard gastroscope could not pass through the oesophagus without resistance.

### Endoscopic treatment

**Figure. 1:**
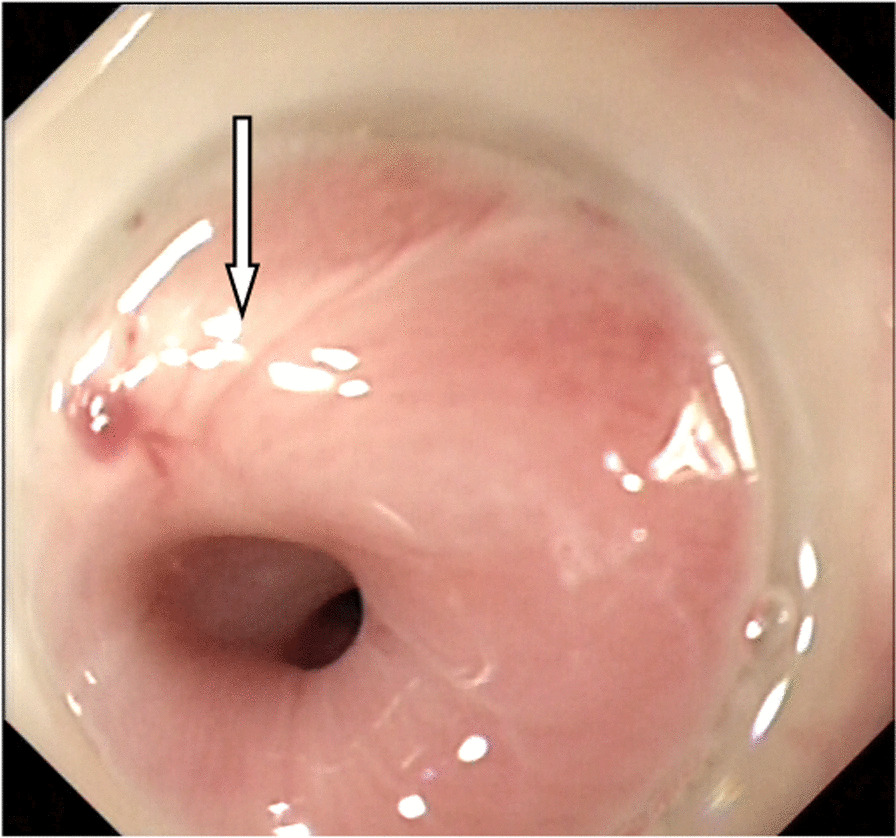
The esophageal stricture was obvious after EIS

**Figure. 2:**
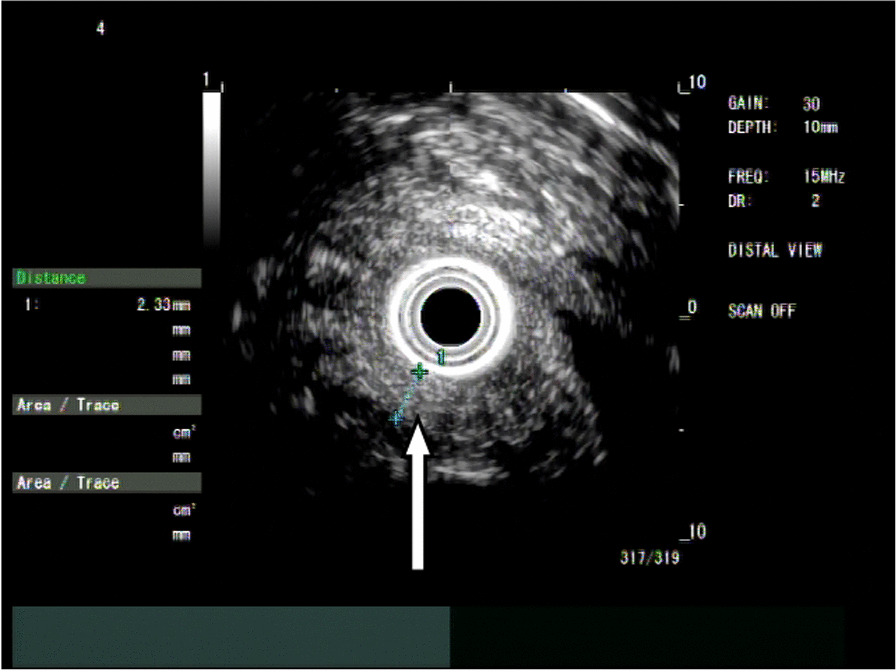
Microprobe of ultrasonic endoscope scaned the scar of esophageal stricture

**Figure. 3:**
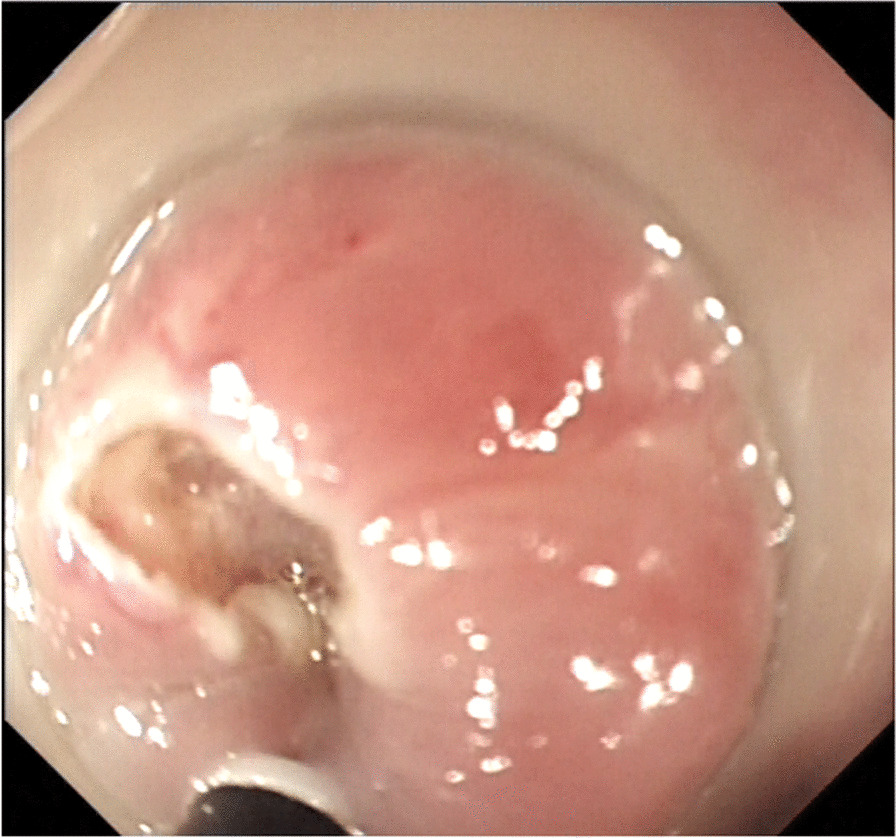
The scar was cut with dualknife according to the measurement by ultrasonic endoscopy

First, we observed retention in the oesophageal cavity through gastroscopy (GIF-HQ290, with insertion tube diameter of 9.9 mm, the inside diameter of 2.8 mm; Olympus Medical Systems, Tokyo, Japan). If the passage of fluid and/or solid was blocked by the stricture, it was necessary to remove the residue. When the oesophageal stricture was fully exposed (Fig. 1), we used the microprobe of ultrasonic endoscope to scan the oesophageal stricture. EUS examination was performed using the microprobe (20 MHz, P2620-M, diameter of 2.6 mm; FUJIFILM, Japan) and high frequency generator (SP-900, FUJIFILM, Japan). Briefly, the procedure was performed with the patient under conscious sedation. The microprobe was inserted through the instrument channel and negotiated across the oesophageal stricture under endoscopic vision. The ultrasonography of the scar manifested as uneven hypoecho, thickening of the mucosa and/or submucosa, and unclear boundary of the oesophageal layers (Fig. 2). Thickness of scar, direction of scar, and distance to incisor were specifically recorded. If residual varices were suspected through the microprobe EUS, we used the doppler EUS to screen the residual varices. Then, we used the dualknife(Model No.KD-650L,Olympus Medical Systems, Tokyo, Japan) to cut the mucosa and submucosa along its longitudinal axis according to EUS measurements (Fig. 3). We took care not to slice the muscularis propria of the oesophagus. The electroincision requires use of incisions with the knife attached to an electrosurgical unit ERBE generator (Elektromedizin GmbH, Tübingen, Germany) with software controlled fractionated cuts in the pure cut of endoscopic submucosal dissection mode (endocut, 60w).

## Result

General data: Sex: There were 7 males and 3 females. Age: The average age was 47 years, ranging from 35 to 59 years. Reason for cirrhosis: There were 9 cases of hepatitis B, and 1 case of alcoholic cirrhosis; Child–Pugh classification: There were 4 cases of grade A, 4 cases of grade B, and 2 cases of grade C. Portal thrombosis: There were 3 cases of combined portal thrombosis. Hepatocellular carcinoma (HCC): There were both 2 cases complicated with HCC. Splenectomy: Three cases were treated with splenectomy. Antiviral: There were 7 cases treated with antivirals. Number of EIS: There were 1–6 times of EIS before. Number of dilation: There were 0 to 4 prior dilations for stricture. Thickness of the scar: The thickness of the stricture scar was 1.1–3.9 mm. Location of scar: The distance to the incisor of the scar ranged from 34 to 39 cm, and the directions of the scar were at one o'clock, two o'clock, nine o'clock, ten o'clock, eleven o'clock, and twelve o'clock. Degree of stricture: There were 3 cases with grade II, 5 cases with grade III, and 2 cases with grade IV (Table 1).

**Table 1:** The general data of the paitents

	Case 1	Case 2	Case 3	Case 4	Case 5	Case 6	Case 7	Case 8	Case 9	Case 10
Sex	F	M	M	M	M	M	F	F	M	M
Age	44	35	58	45	39	59	44	53	56	38
Cirrhosis	HEB	HEB	ALC	HEB	HEB	HEB	HEB	HEB	HEB	HEB
Child–Pugh	B	A	B	A	C	B	B	A	A	C
HCC	−	−	+	−	−	+	−	−	−	−
Portal thrombosis	−	−	−	−	+	−	−	+	+	−
Splenectomy	−	+	−	+	−	−	−	−	+	−
Antiviral therapy	+	−	+	+	−	+	−	+	+	+
EIS treatment/number of times	1	6	3	1	3	2	1	2	2	5
Balloon treatment 4/number of times	4	3	1	0	3	1	4	1	0	0
Degree of stricture before treatment	II	III	IV	II	II	III	III	IV	III	III
Degree of stricture after treatment	O	O	I	O	O	O	I	III	O	I
Direction of scar /o'clock	3	2	10	12	3	11	3	10	9	1
Distance to incisor/centimeter	38	37	34	38	36	39	35	39	36	38
Thickness of scar/millimetre	1.1	3.6	3.2	2.5	1.7	1.9	2.7	3.9	3.1	1.9
Relief of stricture	+	+	+	+	+	+	+	−	+	+

**Figure. 4:**
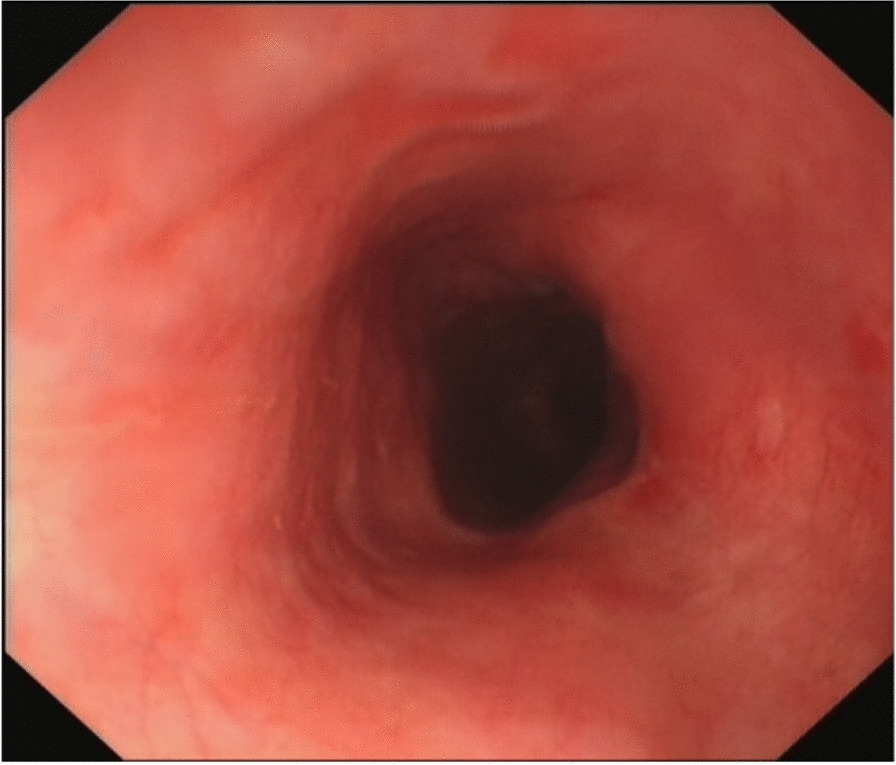
The stricture improved three months after the treatment

F:female; M:male; −:Negative; + :positive; HEB:hepatitis B; ALC:alcohol; HCC:hepatocellular carcinoma; EIS:endoscopic injection sclerotherapy;

Efficacy: The dysphagia was obviously relieved in 9 patients during the follow-up of three to six months (Fig. 4). One patient suffered dysphagia again within one month after the treatment, and endoscopy found that the degree of stricture was grade III. There were no complications of perforation, bleeding or infection.

## Discussion

EIS apparently improves the control of haemorrhage from oesophageal varices and prolongs survival [12, 13], and oesophageal stricture is one of the complications of EIS, with an incidence between 2 and 10% [14]. It seriously affects the quality of life of the patient. The reasons for oesophageal stricture of EIS may be as follows: (1) The vascular endothelium is injured, and chronic inflammation and fibrous scars gradually arise after the sclerosant is injected into the varices. (2) The barrier of the oesophageal mucosa is damaged. (3) The muscularis mucosa and propria of the oesophagus may be injured, which will directly lead to scar stricture. (4) The injection is near the physiological stricture of the oesophagus. (5) The dose of sclerosant is too high. (6) The sclerosant is intensively injected at the same level of the oesophagus, and fibrous scars form at the same circumference of the oesophagus. (7) Repeated injections of sclerosant induce overlape and interlacement of the scar tissue.

The endoscopic treatment for benign oesophageal stricture includes balloon dilation, local incision, stent placement, and endoscopic steroid injection [15–18]. Balloon dilatation mainly achieves its effect through the mechanical tension of the balloon; on the other hand, it can tear the normal mucosa and/or muscularis around the oesophagus. Local incision of oesophageal strictures is reliable [18–21], and will significantly relieve dysphagia. However, We should be alert to the complication of perforation from cutting the muscularis propria of the oesophagus. Stent placement will extend the oesophageal stricture, but, complications from this are much more common, such as chest pain, reflux oesophagitis, displacement or detachment, and tissue embedded stents [21].

Ultrasonic endoscopy can clearly show the five layers of the normal oesophagus [22]. When the oesophageal inflammatory stricture scar is detected through ultrasonic endoscopy, the scar tissue is thicker than the normal mucosa, and the location and depth of the scar can be measured exactly [23, 24].

The particularity of oesophageal stricture after EIS for oesophageal varices is that most patients have risk factors such as residual varices, coagulation dysfunction, and low immunity. This article retrospectively reviews the data of 10 patients with oesophageal stricture of EIS in our hospital treated by cutting the scar through ultrasoound-guided endoscopy. The dysphagia of the stricture was obviously relieved in 9 patients during the follow-up of three to six months, while 1 patient suffered from dysphagia again within one month after the treatment. There were no complications of perforation, bleeding or infection, and some researches reported perforation rate ranges from 0 to 3.5% [25–28] in incisional treatment for esophageal stricture without EUS-guiding.The advantages of ultrasonic endoscopy-guided scar cutting are as follows: (1) It is kept away from residual varices, which avoids bleeding from varices. (2) The scar is cut according to the depth measured by ultrasonic endoscopy, which can reduce the likelihood of perforation. (3) The normal mucosa will not be torn, as can happen in balloon dilatation.

This study has several limitations, mainly related to its retrospective design, few cases, and no longer follow-up. More cases, much more longer follow-up, prospective randomized controlled trial with balloon expansion are needed to evaluate the efficacy of EUS guided cutting scar of esophageal stricture after EIS.

## Conclusion

Ultrasound-guided endoscopy guiding to cut scars is safe and reliable. It may reduce the risk of perforation and bleeding.

## Supplementary Information

**Additional file 1**. The general data of ten paitents.

## Data Availability

All data generated or analysed during this study are included in this published article [and its Additional file 1].

## Abbreviations

EUS: Endoscopic ultrasonography
EIS: Endoscopic injection sclerotherapy
HCC: Hepatocellular carcinoma
HEB: Hepatitis B
ALC: Alcohol
